# Expanding the horizon for breast cancer screening in India through artificial intelligent technologies -A mini-review

**DOI:** 10.3389/fdgth.2022.1082884

**Published:** 2022-12-23

**Authors:** Sudip Bhattacharya, Saurabh Varshney, Petra Heidler, Shailesh K. Tripathi

**Affiliations:** ^1^ Department of Community and Family Medicine, All India Institute of Medical Sciences, Deoghar, India; ^2^ Department of ENT (Otorhinolaryngology), All India Institute of Medical Sciences, Deoghar, India; ^3^ Department for Economy and Health, University for Continuing Education Krems, Krems an der Donau, Austria; ^4^ Department of International Business and Export Management, IMC University of Applied Sciences Krems, Krems an der Donau Austria; ^5^ Department of Health Sciences, St. Pölten University of Applied Sciences, Sankt Pölten, Austria; ^6^ Rajendra Institute of Medical Sciences, Ranchi, India

**Keywords:** artificial intelligence, breast carcinoma, cancer diagnostics, cancer prognosis, computational intelligence

## Abstract

**Introduction:**

Breast cancer is one of the most common cancer among Indian women, with an incidence of 25.8 per 100,000 women according to the Ministry of Health and Family Welfare. Late detection is responsible for poor quality of life (QOL), and it is the leading cause of death. In metropolitan regions, one in every 22 women will have breast cancer over their lifetime; but in rural areas, one in every 60 women will develop breast cancer as per estimates.

**Aim and objective:**

This paper aims to describe the various AI based breast screening technologies which are used in breast cancer screening in India.

**Methodology:**

The literature search was done using “Pub Med,” “Google scholar,” and “Scopus” databases for the key terms “technology,” “cancer research,” “artificial intelligence,” “mammography”, “breast cancer,” “cancer,” and/or “neoplasia in breast.” All the relevant articles were included to support this mini review.

**Results:**

We found that emerging artificial intelligent technologies namely “Niramai”, “iBreastExam,” “MammoAssist” are emerging as an hope for early detection by screening in resource poor settings, in turn, which can improve the QOL among breast cancer patients.

## Introduction

Breast cancer claims the lives of 500,000 women worldwide each year. In India, the annual figure is roughly 75,000 (1). Breast cancer deaths are higher in rural locations than in metropolitan areas due to multiple reasons like delay in diagnosis, cost of treatment, cultural factors and many more (2). According per GLOBOCAN 2020, female breast cancer has surpassed lung cancer as the most commonly diagnosed cancer, with an estimated 2.3 million new cases (11.7%), followed by lung (11.4%), colorectal (10.0%), prostate (7.3%), and stomach (5.6%) cancers (3). Five year overall survival rate of breast cancer in India ranged from 40%–62% (4). Breast cancer is the most common cancer among Indian women, with an incidence of 25.8 per 100,000 women according to the Ministry of Health and Family Welfare (MoHFW) (5).

Late detection is the leading cause of death among breast cancer patients. In metropolitan regions, one in every 22 women will have breast cancer over their lifetime. Government in India is under enormous pressure to address this issue in a cost-effective manner due to huge cancer burden (5). Breast cancer incidence is rapidly increasing in South America, Africa, Europe, and Asia (3).

Approximately, seventy percent of women are diagnosed with cancer in late stages, which has a negative influence on survival rates because of lower likelihood of survival (25%) than early-stage cancers (3). Furthermore, the expense of treating advanced-stage cancer rises by 10–16 times (6). Quality of Life among breast cancer patients can be improved if early detection is achieved by self-breast inspection or mammography. It is also important to down-stage breast cancer (7, 8). Moreover, it is challenging to implement strategies based on self-breast inspection or do mammography in rural regions or low and middle income nations for a variety of reasons, which are- (a) Lack of knowledge, (b). Lack of accessibility for the screening, as only tier 1 or tier 2 cities have screening facilities, (c) Lack of skilled human resources, since the number of available radiologists in India is 1/100,000 population, which is insignificant in comparison to the breast cancer burden, (d) Radiation exposure and pain during the screening (biopsy/FNAC) (e) Technology limitations, because young female's breast tissue is thicker/women have denser breast tissues, the sensitivity of screening tests is quite poor, (f) Cultural barriers are also a concern in rural regions, since rural females are unwilling to test unless the condition is severe, or the cancer is in the advanced stage, (g) Performing mammography is expensive, many rural women avoid this screening procedure, leading to an increase in cancer burden in rural regions (1, 5).

We propose that artificial Intelligence based diagnostic technologies have a potential to revolutionize the scenario for breast cancer screening. So, the current paper aims to explore the extent to which AI based breast cancer screening technologies can be used in India and its future implications.

## Methodology

The literature search was done using “Pub Med,” “Google scholar,” and “Scopus” databases for the key terms “technology,” “cancer research,” “artificial intelligence,” “mammography”, “breast cancer,” “cancer,” and/or “neoplasia in breast.” All the relevant articles were included to support this mini review.

## Results

After literature search, we found these emerging AI based technologies for early detection for the breast cancer. These are:

### iBreastExam

UE Lifesciences created “*iBreastExam (iBE)*,” a “portable, clinically proven, non-invasive, painless, and radiation-free instrument”, that aids in the early diagnosis of breast lesions at the site of contact. The *iBreastExam* or iBE comprises of a portable compression probe with a 4X4 array of piezoelectric tactile pressure sensors, a tablet, and a custom-built electrical board (9).

The *iBE* wirelessly links with a mobile device to show and save data in real time. At the conclusion of each scan, compression data are stored as a unique file on the mobile device and synced to an encrypted database through Dropbox, where a copy is maintained. The *iBE* program does not collect or store personally identifying information, hence anonymity is retained. The *iBE* evaluations are conducted by an ultrasound technician who is trained to operate the *iBE* by the device's manufacturer and the study's primary investigator. Training included one hour of instruction on the device's operations, practice on a breast phantom, and ten *iBE* experiments supervised by the primary investigator. The results of the *iBE* were shown as a pressure map on the touch screen. Green denotes normal breast tissue, but red indicates the detection of a lesion, indicating that the *iBE* recommends additional testing to describe the lesion because it does not discriminate between types of lesions. The patient is in a supine position during the examination. The gadget is calibrated on an unaffected part of the breast, and it may be recalibrated several times. The *iBE* aggregates the collected data into a 4X4 array map of the breast, with each square representing the 4X4 array of the piezoelectric finger sensor (PEFS). This map was split into sectors delineated by three consecutive hours of a clock to provide a direct comparison with the clock positions assigned by mammography or ultrasonography (9).

The total procedure consists of four phases.

*Step-1 Tissue differentiation*-The differences in tissue elasticity are assessed as breast lumps (lesions) are stiffer than healthy breast tissue due to tissue differentiation.
*Step-2 Tactile Imaging-* iBreastExam's Dynamic Co-Planar Capacitive Sensors assess these changes digitally, in real-time, and non-invasively.
*Step-3 Identification*-iBreastExam standardizes with minimum training the detection of breast lumps.
*Step-4 Documentation-* Quick and painless recording of routine breast examinations inform and empowers care professionals and women.

A clinical trial (NCT02814292) was conducted to assess the sensitivity of the iBE in women undergoing diagnostic breast imaging. Eligible patients were adults who consulted a breast imaging center for a diagnostic evaluation. After undergoing an *iBE* examination done by a skilled ultrasound technician, patients had their prescribed imaging. The data included demographic, imaging, and biopsy information. A total of seventy-eight *iBE* examinations were taken, by 77 females and one man, with a mean age of 42 (21–79). All patients had ultrasound evaluation, 52 underwent diagnostic mammography, and 39 underwent biopsies. In 60 individuals, imaging and/or biopsy verified the presence of a tumor (fibroadenoma, cyst, papilloma, myofibroblastoma, fat necrosis, DCIS, or malignancy). Twelve people were diagnosed with cancer. In all, 342 quadrants were scanned, 77 quadrants had lesions validated by imaging, and iBE accurately detected 66 lesions, achieving a sensitivity of 86% and a specificity of 88%. This validation research confirmed the high sensitivity of *iBE* in detecting clinically relevant lesions in individuals undergoing diagnostic imaging (10). Another research conducted in Bangalore compared *iBE* to clinical breast examination, mammography, and breast ultrasonography using a triple-blind design. A total of 1,019 Indian women who visited a tertiary hospital for a yearly health check were examined. Each woman was assessed by three separate, blinded methods: iBreastExam (iBE), Clinical Breast Examination (CBE) performed by a trained physician, and Breast Imaging (mammography or breast ultrasound). Imaging indicated that 93 of 916 registered patients had at least one breast lesion. iBreastExam revealed Sn_1/4_ 84%, Sp_1/4_ 94%, PPV _1/4_ 60%, and NPV _1/4_ 98%, whereas Clinical Breast Examination demonstrated Sn_1/4_ 65%, Sp_1/4_ 94%, PPV _1/4_ 52%, and NPV _1/4_ 96%. iBE found breast tumors with Sn_1/4_ 85%, Sp_1/4_ 93% in 314 women less than 40 years old. iBE recognized all malignant lesions, whereas clinician CBE missed one non-palpable malignant lesion. The study revealed that the point-of-care Breast Imaging equipment (iBreastExam) was 19% more sensitive than CBE at detecting breast lesions, with excellent specificity (94%) and negative predictive value (NPV) (98%) (11).

In third research, the intelligent Breast Exam (iBE) was compared to the usual Clinical Breast Exam (CBE) and mammography in a western screening population. In this research, women coming for screening or diagnostic workup got iBE followed by CBE and finally mammography. The classification of mammography as negative (BI-RADS 1 or 2) or positive (BI-RADS 3, 4, or 5). Calculations of accuracy and score were conducted (9). The study indicated that 516 women were enrolled between April 2015 and May 2017. A total 486 patients completed the iBE, CBE, and mammogram. There were 101 positive findings for the iBE, 66 positive results for the CBE, and 35 positive mammograms. iBE and CBE showed considerable agreement regarding classification (Kappa = 0.53), but little agreement with regard to mammography (Kappa = 0.08). Specificity was 80.3% and negative predictive value was 94% for iBE. Only five out of 486 individuals in this group had a cancer; iBE and CBE detected three of these five. Both modalities missed two tiny cancers: a 3-mm retro-areolar and a 1-cm axillary tail. The study found that iBE and CBE function comparably as triage tools. iBE missed just a few breast tumors found with mammographic screening. In conclusion, the findings revealed that iBE and CBE can collaborate as triage techniques to considerably minimize the percentage of patients in resource-limited settings who require further diagnostic imaging (9).

### Mammoassist

As a part of the “Make it in India” initiative, Telerad Tech, a global healthcare technology from Bengaluru, has introduced “MammoAssist”, a new AI-powered tool that detects early-stage breast cancer. “MammoAssist” analyses mammograms and processes imaging data to identify radiologic signs of early-stage breast cancer and categorises it for BIRADS Scoring using a fully organised template. This template is then sent to a radiologist, who confirms and validates the mammography results before issuing the formal report. “MammoAssist” has been proved in trials to increase a radiologist's efficiency and output by over 50%. MammoAssist can assist in early detection of breast cancer and start generating a comprehensive report including all substantial radiologic analyses such as “Microcalcification,” “Macrocalcification”, “Clustered Calcification,” “Lesions & Lymph Node,” “Architectural Distortion,” “Bilateral Asymmetry,” “Breast Parenchymal Structure,” “Dimensions,” “Form,” “Position,” and “Intensity analysis” with BI-RADS score. It improves a radiologist's ability to investigate patients using excellent precision, as well as providing a standard interface for communicating with healthcare systems such as Radiology Information System-Picture Archiving and Communications System (RIS-PACS) and “Electronic Medical Records” using industry standard “APIs” and protocols.

MammoAssist may create reports in “English,” “Spanish,” “German,” “French,” “Italian,” “Polish,” and “Portuguese,” among other languages. Telerad Tech's Latest Generation Ai - powered (RIS-PACS) Platform “RADSpa” is powered by “MammoAssist.” “RADSpa” has also been used to analyse over 20 million studies and is implemented in over 25 countries. “RADSpa” is FDA approved and CE certified. “MammoAssist” is a cost-effective answer to the above-mentioned developing problem, as it serves as a catalyst for radiologists to evaluate mammograms in the shortest amount of time possible. It has the potential to lower the expenses of mass screening programmes (12).

Additionally, scientists from Guwahati's Institute of Advanced Study in Science and Technology (IASST) have devised a deep-learning-based (artificial intelligence) approach to assess hormone levels for breast cancer prediction. This method will aid in the early diagnosis of cancer. With the use of immunohistochemistry, Lipi B Mahanta, the director of a research group at the IASST's Central Computational and Numerical Sciences Division, has created a revolutionary deep-learning-based quantitative assessment of the hormone oestrogen or progesterone Immunu Histo Chemistry (IHC). Antibodies are used in IHC to look for specific antigens (markers) in a sample tissue. It essentially “colours” the antibodies that bind to the antigen, allowing the antigen to be examined under a microscope. Then comes deep learning, which aids in the interpretation of pictures to identify (or segment) malignant cells. The programme was created using data from B Borooah Cancer Institute. According to a news release from the Department of Science and Technology, the parent body of IASST, “the suggested architecture, namely IHC-Net, can meaningfully separate the exact positive and negative nuclei from tissue pictures.” With 98 percent accuracy, these algorithms can categorise and identify malignant cells in smear pictures. Mahanta used the same approach for the identification of oral cancer in another investigation (13).

### Niramai

According to a literature review, two startups, UE Life Sciences and Non-Invasive Risk Assessment with Machine Intelligence” (Niramai), can accurately identify breast cancer using artificial intelligence (14). In 2016, Niramai Health Analytics (Bangalore) developed a non-invasive, low-cost breast cancer screening method based on the tracking of body heat embedded with artificial intelligence (Thermalytix) technology (14). The SMILE-100 System, a unique radiation-free, non-contact, and accurate breast cancer screening solution in India, has gained US FDA authorization for its first device (14). The SMILE-100 System enables healthcare providers to make more informed decisions regarding breast cancer screening and diagnosis by facilitating the visualization of high thermal activity patterns on thermal imaging as hotspots. SMILE-100's usage of their proprietary artificial intelligence-based algorithms to verify the quality of input thermal pictures can significantly minimize mistakes in thermal image collection and enable low-skilled health professionals to undertake imaging with confidence (14). The thermal analysis of skin temperature distribution, with the purpose of acquiring information on a probable inside tumor, has greater benefits for indicating aberrant metabolism in the early stages of cancer (15). Consequently, thermography is a useful tool for localizing alterations in blood perfusion that may be caused by inflammation, angiogenesis, or other factors that may induce temperature distribution asymmetry. These asymmetries, as well as the existence of hot and cold temperature zones, are a reliable sign of an underlying issue (16). Even though mammography and ultrasound diagnostics are normally conducted manually by specialists, there is a great desire for automated approaches that offer an objective answer that might be utilized as a second opinion (17). Some automated approaches rely on the analysis of thermograms by separating the picture into segments of interest and then analysing each segment individually. Henceforth, the segmentation of pictures refers to the process that splits a digital image into numerous pieces and is typically used to discover regions of interest and information-rich elements in digital images (18). The comparison of thermal asymmetries between the left and right breasts is one of the most important methods for distinguishing breast disorders. This method can detect cancer cells five years before mammography and other invasive testing. It is a non-contact method. This approach uses technology that is portable and one-tenth the expense of traditional mammography (3,500–5,000 INR) (14).

Moreover, the expertise level required to operate this equipment is not especially complicated. The gadget is extremely easy for nurses and paramedics to operate. This technique enables remote radiological consultations *via* teleradiology. Therefore, it cuts hospitals' full-time personnel costs. This technique has been tested on over 500,000 women in over 60 hospitals/diagnostic centers and over 2,000 screening camps. It assisted in the early diagnosis of cancer in certain individuals. The patient enters a room and receives instructions from outside throughout this evaluation. The examination is performed in strict confidence. Later, it was determined that the privacy element was particularly important for rural women. Consequently, this method has achieved great adoption.

In a multicentric investigation (*n* = 470), it was shown that the test had a sensitivity of 91.02% (95% CI, 81.8% to 96%), a specificity of 82.39% (95% CI, 78.2% to 86%), and a negative predictive value (NPV) of 97.88% in detecting breast cancer. In asymptomatic screening populations, sensitivity and specificity were 100% and 92.41%, respectively. Sensitivity was 89.85% and specificity was 69.04% in symptomatic women" (14).

### Mammoalert

MammoAlert is another AI-based method for breast cancer screening; it uses an immunoassay that operates on Pandora CDx technology and generates results in 15 min from a single blood drop. Pandora CDx employs a unique sedimentation technique to conduct sandwich immunoassays with beads. In the sample loading chamber, the disk includes fluorescently labelled “detection” antibodies and beads bearing “capture” antibodies. In the separating zone, the disk also contains density centrifugation medium. After loading the sample, it is incubated for a few minutes. The disk is then spun to extract unreacted reagents and sample from the beads. The sedimented beads' fluorescence signal is detected using an optical detector. In the sample, four protein markers associated with breast cancer are analyzed. According to the research, an algorithm has also been built using deep machine learning and AI to estimate the likelihood that a person has breast cancer. The results are available in less than 30 min and are transmitted to the test operators, physician, and patient through a smartphone application (19).

*MammoAlert* is a test that may be performed in any rural region or slum, at a minimal cost, and perhaps early. It has a specificity and sensitivity of greater than 85 percent, compared to 70 percent for mammography in India, and costs less than $5 each test (19). While the precise location of the tumour must be confirmed by mammography or other methods, this technique has the potential to improve QOL of millions of women worldwide by detecting breast cancer earlier. Although this looks to be promising, further research is required to demonstrate that *MammoAlert* is useful to patients. According to Saxena, *MammoAlert* has been examined in eight tests involving over one thousand samples. However, the results have not yet been published and are therefore unavailable to the public. According to Lopes, *MammoAlert* is in an even earlier stage of development than *Niramai.* This is a blood-based diagnostic, and research is currently being conducted on these tests (19).

## Discussion

The technology acceptance model (TAM) is a theory which describe how a technology is adapted by a certain population. An element that motivates people to use technology is their behavioural intention. The attitude (A), which is the broad perception of the technology, influences the behavioural intention (BI).

When people are confronted with new technology, the model describes that many variables influence their decision on utilization: Two main factors are- Perceived usefulness (PU) – As per Fred Davis (1989) “the extent to which a person feels that utilizing a certain system would improve their work performance.” It refers to when people believe that technology is suitable for their needs and the second is the Perceived ease-of-use (PEOU). Davis defines this as “the degree to which a person feels that utilizing a specific system would be painless” (Davis 1989). It is also mentioned that if the technology is simple then it can be adopted very quickly (20).

External socio-cultural factors play a significant role in determining their attitude and actual utilization, i.e., the ultimate endpoint. The same may be said about the acceptance of new technologies in the healthcare field.

Breast cancer screening technology is improving every day. It began with self-breast inspection, then progressed to x-ray and mammography, and is now detectable utilising artificial intelligence embedded with different thermolytic methods such as Niramai, MammoAlert, IHC-Net, and iBreastExam. In future many technologies may evolve for early detection of the breast cancer (Table 1).

**Table 1:** Ai based technologies used for early detection of breast cancer.

Name of the technology	Mode of working	Research conducted and latest findings	Advantages
“iBreastExam (iBE)”	Components- It is a Hand-held device. The iBreastExam or iBE comprises of a portable compression probe with a 4X4 array of piezoelectric tactile pressure sensors, a tablet, and a custom-built electrical board. Working- The iBE wirelessly links with a mobile device to show and save data in real time. At the conclusion of each scan, compression data are stored as a unique file on the mobile device and synced to an encrypted database through Dropbox, where a copy is maintained. The results of the iBE were shown as a pressure map on the touch screen. Green denotes normal breast tissue, but red indicates the detection of a lesion, indicating that the iBE recommends additional testing to describe the lesion because it does not discriminate between types of lesions. The patient is in a supine position during the examination. The gadget is calibrated on an unaffected part of the breast, and it may be recalibrated several times. Interpretation- The iBE aggregates the collected data into a 4X4 array map of the breast, with each square representing the 4X4 array of the PEFS. This map was split into sectors delineated by three consecutive hours of a clock to provide a direct comparison with the clock positions assigned by mammography or ultrasonography (9).	Research type-Clinical trial ((NCT02814292) Research findings- A total of seventy-eight iBE examinations were taken, by 77 females and one man, with a mean age of 42 years. (21–79). All patients had ultrasound evaluation, 52 underwent diagnostic mammography, and 39 underwent biopsies. In 60 individuals, imaging and/or biopsy verified the presence of a tumor (fibroadenoma, cyst, papilloma, myofibroblastoma, fat necrosis, DCIS, or malignancy). Twelve people were diagnosed with cancer. In all, 342 quadrants were scanned, 77 quadrants had lesions validated by imaging, and iBE accurately detected 66 lesions, achieving a sensitivity of 86% and a specificity of 88%. This validation research confirmed the high sensitivity of iBE in detecting clinically relevant lesions in individuals undergoing diagnostic imaging (10). Research type-Clinical trial (Triple blinded) Research findings- A total of 1,019 Indian women who visited a tertiary hospital for a yearly health check were examined. Each woman was assessed by three separate, blinded methods: iBreastExam (iBE), Clinical Breast Examination (CBE) performed by a trained physician, and Breast Imaging (mammography or breast ultrasound). Imaging indicated that 93 of 916 registered patients had at least one breast lesion. iBreastExam revealed Sn1/4 84%, Sp1/4 94%, PPV 1/4 60%, and NPV 1/4 98%, whereas Clinical Breast Examination demonstrated Sn1/4 65%, Sp1/4 94%, PPV 1/4 52%, and NPV 1/4 96%. iBE found breast tumors with Sn1/4 85%, Sp1/4 93% in 314 women less than 40 years old. iBE recognized all malignant lesions, whereas clinician CBE missed one non-palpable malignant lesion. The study revealed that the point-of-care Breast Imaging equipment (iBreastExam) was 19% more sensitive than CBE at detecting breast lesions, with excellent specificity (94%) and negative predictive value (NPV) (98%) (11). Research type-Validation study (output indicator = Kappa statistics) Research findings- The study indicated that 516 african women were enrolled between April 2015 and May 2017. A total 486 patients completed the iBE, CBE, and mammogram. There were 101 positive findings for the iBE, 66 positive results for the CBE, and 35 positive mammograms. iBE and CBE showed considerable agreement regarding classification (Kappa = 0.53), but little agreement regarding mammography (Kappa = 0.08). Specificity was 80.3% and negative predictive value was 94% for iBE. Only five out of 486 individuals in this group had a cancer; iBE and CBE detected three of these five. Both modalities missed two tiny cancers: a 3-mm retro-areolar and a 1-cm axillary tail. The study found that iBE and CBE function comparably as triage tools. iBE missed just a few breast tumors found with mammographic screening. In conclusion, the findings revealed that iBE and CBE can collaborate as triage techniques to considerably minimize the percentage of patients in resource-limited settings who require further diagnostic imaging (9).	• Clinically validated • Non-invasive • Painless • Radiation-free device • FDA approved
“*MammoAssist”-RADspa platform*	Technology used- It is a teleradiological software. It works on the principle of deep Learning and image processing. Working-“MammoAssist” analyses mammograms and processes imaging data to identify radiologic signs of early-stage breast cancer and categorises it for BIRADS Scoring using a fully organised template. This template is then sent to a radiologist, who confirms and validates the mammography results before issuing the formal report.	Research type-Clinical trial Research findings-“MammoAssist” has been proved in trials to increase a radiologist's efficiency and output by over 50%. MammoAssist can assist in early detection of breast cancer and start generating a comprehensive report including all substantial radiologic analyses such as “Microcalcification,” “Macrocalcification”, “Clustered Calcification,” “Lesions & Lymph Node,” “Architectural Distortion,” “Bilateral Asymmetry,” “Breast Parenchymal Structure,” “Dimensions,” “Form,” “Position,” and “Intensity analysis” with BI-RADS score. It improves a radiologist's ability to investigate patients using excellent precision, as well as providing a standard interface for communicating with healthcare systems such as Radiology Information System-Picture Archiving and Communications System (RIS-PACS) and “Electronic Medical Records” using industry standard “APIs” and protocols.	• Helps in cancer diagnosis with >50% more accuracy than mammogram
Deep-learning-based -Immuno Histo Chemistry (IHC) net	Technology used- Deep-learning-based quantitative assessment of the hormone oestrogen or progesterone Immunu Histo Chemistry (IHC). Working- Antibodies are used in IHC to look for specific antigens (markers) in a sample tissue. It essentially “colours” the antibodies that bind to the antigen, allowing the antigen to be examined under a microscope. Then comes deep learning, which aids in the interpretation of pictures to identify (or segment) malignant cells.	Research findings- 1. A new fully convolutional neural network architecture is proposed for segmentation. 2. Evaluation of proportion score of the Allred scoring system is done. 3. 40 novel IHC whole slide images split into 20 training and 20 tests were used. 4. U-net, Segnet, and FCN-8 were also applied and compared on the IHC dataset. 5. New architecture outperforms the three state-of-the-art segmentation methods. 6. Final result gives intensity score evaluation with the highest accuracy (99.11%). 7. All results were corroborated statistically.	• This method can identify malignant cells with 98% accuracy
*MammoAlert*	Technology used - Pandora CDx technology Working-Pandora CDx employs a unique sedimentation technique to conduct sandwich immunoassays with beads. In the sample loading chamber, the disk includes fluorescently labelled “detection” antibodies and beads bearing “capture” antibodies. In the separating zone, the disk also contains density centrifugation medium. After loading the sample, it is incubated for a few minutes. The disk is then spun to extract unreacted reagents and sample from the beads. The sedimented beads’ fluorescence signal is detected using an optical detector. In the sample, four protein markers associated with breast cancer are analyzed. According to the research, an algorithm has also been built using deep machine learning and AI to estimate the likelihood that a person has breast cancer.	Research findings- *MammoAlert has been examined in eight tests involving over one thousand samples. However, the results have not yet been published and are therefore unavailable to the public.*	• Just requires a finger prick • The findings are accessible in less than 30 min • Can be sent to the test operators, doctor, and patient *via* a smartphone app • It can be performed in any rural community or slum areas • It is very low cost (5$/ test) • Early detection (than Niramai) Specificity > 80% • Sensitivity > 85%, as compared to 70% for mammography
“Niramay-Thermalytix”	Technology used- Thermalytix Working- The thermal analysis of skin temperature distribution (cancer and non-cancer area), with the purpose of acquiring information on a probable inside tumor, has greater benefits for indicating aberrant metabolism in the early stages of cancer (15). Consequently, thermography is a used for localizing alterations in blood perfusion that may be caused by inflammation, angiogenesis, or other factors that may induce temperature distribution asymmetry. These asymmetries, as well as the existence of hot and cold temperature zones, are a reliable sign of an underlying issue (16). The segmentation of pictures refers to the process that splits a digital image into numerous pieces and is typically used to discover regions of interest and information-rich elements in digital images (18). The comparison of thermal asymmetries between the left and right breasts is one of the most important methods for distinguishing breast disorders.	Research findings- (Full data not available in public domain) In a multicentric investigation (*n* = 470), it was shown that the test had a sensitivity of 91.02% (95% CI, 81.8% to 96%), a specificity of 82.39% (95% CI, 78.2% to 86%), and a negative predictive value (NPV) of 97.88% in detecting breast cancer. In asymptomatic screening populations, sensitivity and specificity were 100% and 92.41%, respectively. Sensitivity was 89.85% and specificity was 69.04% in symptomatic wome." (14).	• Noninvasive • Low cost (3000-5,000 INR) • No skilled manpower required • Sensitivity- 91.02% (95% CI, 81.8% to 96%) • Specificity- 82.39% (95% CI, 78.2% to 86%) • Net Positive predictive Value- 97.88% • FDA approved

## Disadvantages of AI based breast cancer screening

Despite of benefits of newer technologies, new AI-based devices, such as i-BreastExam, have limits. In most institutions in high-income nations, screening for breast cancer begins at age 40 or 50. Obviously, the current research covered women aged 40 or older; nevertheless, the optimal age to begin breast cancer screening in Nigerian women is unclear. Breast cancer incidence appears to drop with age, and about one-third of African women with breast cancer are younger than 40 years; thus, adopting the cut-off age of 40 years or older in most centres in LMICs might overlook up to thirty percent of African women with breast cancer. Although a comparison between dense and non-dense breasts may give insight into the likely performance of the iBreastExam in women younger than 40 years, it would be good to understand the influence of young age, breast density, and minor lesions on iBreastExam performance (21).

## Window of opportunity

Under the auspices of National Program for Prevention and Control of Cancer, Diabetes, CVD and Stroke (NPCDCS), the MINISTRY OF HEALTH & FAMILY WELFARE, GOVERNEMNT OF INDIA, is delivering cancer-related health education services. The detection of early warning signs of common cancer, for example, is performed at the subcentre and primary health centre levels. At the level of the district, “opportunistic” screening of common malignancies (oral, breast, cervix, and prostate) is ongoing, and self-breast examination (SBE) is used for breast cancer screening (22). Multiple studies on AI-based breast cancer screening have shown that untrained health-care workers may successfully utilize AI-based devices like iBreastExam with minimum training and greater sensitivity than clinical breast examination. The iBreastExam can alleviate the problem of a lack of specialists for early detection of breast cancer in Indian communities where cultural barriers are prevalent, thereby demonstrating the potential to delay the progression of advanced-stage breast cancer and lead to an improvement in the quality of life for cancer patients through early detection. But the main problem is the excessive reliance on high-tech, high-cost screening, which is not always beneficial for poor nations like India with underdeveloped and low funded healthcare systems. It is also likely that at least some overtreatment bias will arise, adding another strain to the healthcare system. In addition to this, the initial investment costs will increase if such technologies are used for breast cancer screening. As an alternative, we suggest that these new technologies can be utilized in public-private partnership model models along with conventional techniques like SBE/mammography in government hospitals. Thus, additional (Indian) data may be gathered, and the government can fund/implement new AI based screening technologies/devices under NPCDCS programme in the future after doing a cost-benefit and cost-effectiveness analysis. So, whenever we try to devise or implement a new technology, we should consider the socio-cultural feasibility, ethical and economic aspects as well as the mindset of the people for mass screening. Additionally, health education will continue to pay a vital role in breast cancer screening. This way, quick adoption of healthcare technology takes place among all segments in our society.

## Limitations

There is a restriction to this evaluation. It is a narrative review in which the evidence is retrieved and synthesized without using a systematic technique. We used a negative review technique due to the scarcity of literature in this field particularly in India, which is still a problem. Further research needs to be done and findings contrasted with the results from Africa, America and Europe.

## Conclusion

We concluded that AI based breast cancer screening is important to make a better scenario for patients having breast cancer with a goal to down stage the breast cancer and improve the quality of lives among the patients having breast cancer.
